# Protein Network Studies on PCOS Biomarkers With S100A8, Druggability Assessment, and RNA Aptamer Designing to Control Its Cyst Migration Effect

**DOI:** 10.3389/fbioe.2020.00328

**Published:** 2020-05-13

**Authors:** Subramaniyan Manibalan, Ayyachamy Shobana, Manickam Kiruthika, Anant Achary, Madasamy Swathi, Renganathan Venkatalakshmi, Kandasamy Thirukumaran, K. Suhasini, Sharon Roopathy

**Affiliations:** ^1^Centre for Research, Kamaraj College of Engineering and Technology, Madurai, India; ^2^Department of Biotechnology, Kamaraj College of Engineering and Technology, Madurai, India

**Keywords:** network analysis, druggability, RNA aptamer, lim method, pcos targets, protein network

## Abstract

The prevalence of polycystic ovary syndrome (PCOS) has been gradually increasing among adult females worldwide. Laparoscopy drilling on ovary is the only available temporary solution with a high incidence of reoccurrence. S100A8 with S100A9 complex is believed to facilitate the cyst migration in PCOS condition. The high evident protein interaction network studies between PCOS biomarkers, cancer invasion markers, and the interactors of S100A8 confirm that this protein has strong interaction with other selective PCOS biomarkers, which may be associative in the immature cyst invasion process. Through the network studies, intensive structural and pathway analysis, S100A8 is identified as a targetable protein. In this research, the non-SELEX *in silico* method is adapted to construct RNA Library based on the consensus DNA sequence of Glucocorticoid Response Element (GRE) and screened the best nucleotide fragments which are bound within the active sites of the target protein. Selected sequences are joined as a single strand and screened the one which competitively binds with minimal energy. *In vitro* follow-up of this computational research, the designed RNA aptamer was used to infect the MCF7 cell line through Lipofectamine 2000 mediated delivery to study the anti-cell migration effect. Wound Scratch assay confirms that the synthesized 18-mer oligo has significant inhibition activity toward tumor cell migration at the cellular level.

## Introduction

Nucleotide aptamers are successfully explored as better therapeutics to treat diseases and disorders. Time-consuming low-throughput procedures have been in practice to design the aptamers *in vitro* (Ghavami et al., 2009). Therefore, *in silico* non-SELEX approach is the better choice to perform the selection of aptamers, which involves the construction of an oligonucleotide library without amplification and binding them with suitable target protein unlike SELEX (Berezovski et al., 2006; Tseng et al., 2011). Designing the RNA aptamer for the validated biomarker helps us to normalize the disease state at the genetic level. Hence, delivering a well-designed aptamer against response elements (REs) can control the strange translation of the target gene. REs are the critical elements involved in the activation of target gene regulation. Inhibiting biomarkers of specific pathophysiological conditions at the molecular level is a better choice to oversee the disease (Strimbu and Tavel, 2010). Target validation is one of the necessary procedures in drug discovery protocol. Since the exact cause of polycystic ovary syndrome (PCOS) is imprecise (Sir-Petermann et al., 2002), it is tedious to identify the best target clinically. Assay on endometrial cell migration is one of the diagnostic tools to identify the complications of this syndrome, and metformin has a proven attenuating effect on the invasion of endometrial cells of diseased women (Tan et al., 2011). Previously, researchers have found that 500 biomarkers are prevalent in PCOS (Dai and Lu, 2012). In this research work, we focused on S100A8 protein which is one of the important biomarkers in PCOS. Protein–protein interaction network (PPIN) is used to identify the associative proteins and its pathways in PCOS. Additionally, druggable properties of S100A8 were studied through pocket analysis. Besides, the aptamer library for specific RE of S100A8 was constructed by a non-SELEX fragment approach. The best aptamer sequence was screened through quality assessments, such as affinity and stability parameters.

## Materials and Methods

### Network Profile of S100A8 in Polycystic Ovary Syndrome and Enrichment Analysis

Interactors of S100A8 are obtained from BioGRID^3^.^5^, a dataset repository (Oughtred et al., 2019), and the molecular interaction network was constructed in STRING Database (Szklarczyk et al., 2019). Biomarkers specific to PCOS and cancer cell invasion are retrieved from the recent research articles (Daan et al., 2016; Lu et al., 2017; Gerashchenko et al., 2019) and are used to construct another network. Both the networks were merged to find the first shell interactors of S100A8. Cytoscape 3.7.2 is employed to merge the networks and find proteins which are associated with S100A8. Pathways of S100A8 and its clusters are identified by using ClueGO, a Cytoscape application for clustering the functional network by terms or pathways (Bindea et al., 2009). Molecular functions of Gene Ontology (GO), Reactome Pathway Database (Croft et al., 2011), and the Kyoto Encyclopedia of Genes and Genomes (KEGG) pathways are used as resources for enrichment analysis. *P*-value 0.005 is set as a significance to select the clusters of S100A8.

### Structure and Druggability Studies on S100A8

Druggability analysis is used to predict the receptiveness and stability of drug target. Physiochemical and geometric properties such as number of pockets, druggable score, and pocket volume determine the efficiency of the target candidate. DoGSite Scorer is used for binding site prediction analysis and druggability assessment, which is based on heavy-atom coordinates employing support vector machines (SVMs) (Volkamer et al., 2012). Pocket volume, lipophilic character, and pocket enclosures were accounted for simple score calculation to suggest the competence of targetability. Three-dimensional structure of the target was retrieved from PDB (ID: 5HLV) and used for the druggable screening.

### Glucocorticoid Response Elements for S100A8

REs are the inducers of the receptor and ligand interaction which results in the expression or activation of a particular protein. Since the aptamers are crucial elements in the control of target expression so we decide to design RNA oligomer against specific PCOS targets. Glucocorticoid RE (GRE) (Hsu et al., 2005), hypoxia RE (HRE) (Rees et al., 2001), antioxidant RE (ARE) (Nioi et al., 2003), and interferon gamma (INF-γ) RE (IRE) (Yang et al., 1990) are identified as the influencing REs of the S100A8 gene. Specifically, GREs have the proficiency to inhibit S100A8 through the downregulation of leukocyte transmigration. Glucocorticoids also induce the expression of inhibition factor for macrophage migration, which ultimately downregulates the cyst inflammation. The earlier research report shows that GRE consists of two half-sites with three spacer bases; the consensus pseudo palindromic sequence of GRE is 5′ CAGAACA**TCA**TGTTCTGA 3′ (Weikum et al., 2017).

### Nucleotide Fragment Library Construction

RNA Composer utilizes the Dot-Bracket format notation of the secondary structure sequence to model the RNA, and the 3D element of modeled RNA was chosen from RNA Frabase (Biesiada et al., 2016). RE is a sequence, which binds with the receptor and plays a crucial role in expression, so the RNA analog library of specific RE was created to mimic the inhibitory action. The consensus sequence was segregated as fragments in such a way that six nucleotides at a stretch were taken per fragment (Figure 4A) for analog library construction. Resulted library sequences were later utilized for binding studies with the target by RNA-Lim method and recognized the various conformations of fragments bound in the active sites of the protein (Hall et al., 2015). Fragments which bound on active sites are selected to design the high précised aptamer model. Diversity in the exhibited conformations of ssRNA–protein complexes was meticulously sampled to construct a fragment library. MC-Fold | MC-Sym pipeline was employed to obtain the secondary and tertiary structures of the constructed aptamer (Parisien and Major, 2008). The proposed mechanism for PCOS control through the aptamer binding on S100A8 is illustrated in Figure 2. Refinement on docking results of their chain-forming poses was done in Molecular Operating Environment (MOE) suit (Ahirwar et al., 2016). The fragment-based approach was adopted for competent docking with S100A8 (Ahirwar et al., 2016); this unusual method has numerous advantages over conventional rigid based docking.

### Affinity and Stability Studies of the Designed Aptamer

PatchDock tool is efficiently used to study the binding properties of designed aptamers with target protein (Schneidman-Duhovny et al., 2005). Based on the global binding energy, FireDock is employed to decipher the docked result by flexible refinements rather than the rigidity of protein and also it optimizes the side-chain residues, which minimizes the rigid body conformation of the interactive protein (Mashiach et al., 2008). Previously, it was reported that the stability of RNA will be analyzed by the inverted repeats which form stable hairpin loops (Ahirwar et al., 2016). Oligoanalyzer is an inclusive oligonucleotide scrutinizer employed to check the hairpin loop and stability of the designed aptamer (Owczarzy et al., 2008).

### Anti-migration by Scratch Wound Healing Assay on Cell Lines

MCF-7 cells were seeded into a 24-well tissue culture plate containing antibiotic-free minimal essential medium (MEM) and incubated for 24 h at 37°C with 5% CO_2_. Sterile microtip was used to a make scratch on the 80% confluence monolayer (Camorani et al., 2014). The culture medium was immediately replaced with fresh medium to remove the dislodged cells. Then, 20 nmol/l of the designed aptamer with the transfecting agent, Lipofectamine 2000 (Invitrogen), was dissolved in dimethyl sulfoxide (DMSO) for timeline studies (Zhou et al., 2008). Cell migration of both sample and control were monitored and compared to study the aptamer effect on cell migration.

## Results and Discussion

### S100A8 Network With Polycystic Ovary Syndrome Biomarkers

**FIGURE 1:**
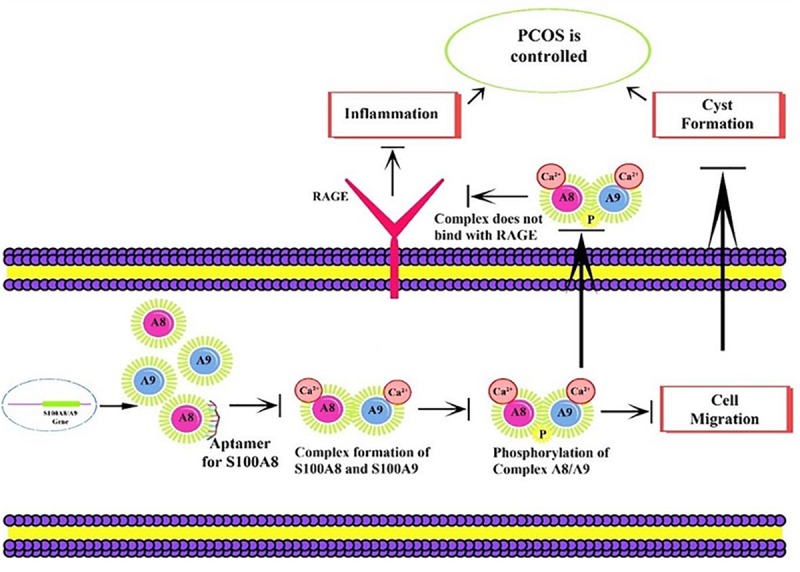
Proposed mechanism of aptamer inhibition. Aptamer binds to the S100A8 and prevents the intracellular initial complexation with S100A9. Due to aptamer binding, extracellular receptor of advanced glycation end products (RAGE) complexed inflammatory sequences are also prevented.

S100A8 interacts with 74 proteins (Supplementary File S1). The high confidential STRING network of S100A8 interactors has 55 nodes with 181 edges. The second network with biomarkers has 98 nodes with 431 edges; among 98, two proteins (SLC35D2, MORC4) are unconnected with the main network. Interactor network and biomarker network details are given in Supplementary Material. Merged network with connected nodes of the interactors and the biomarkers shows 96 nodes with 430 edges (interactions) that are illustrated in Figure 1. Immediate interacting proteins with s100A8 are shown in yellow. By the network analysis, we found that S100A8 directly interact with 10 proteins (Table 1).

**FIGURE 2:**
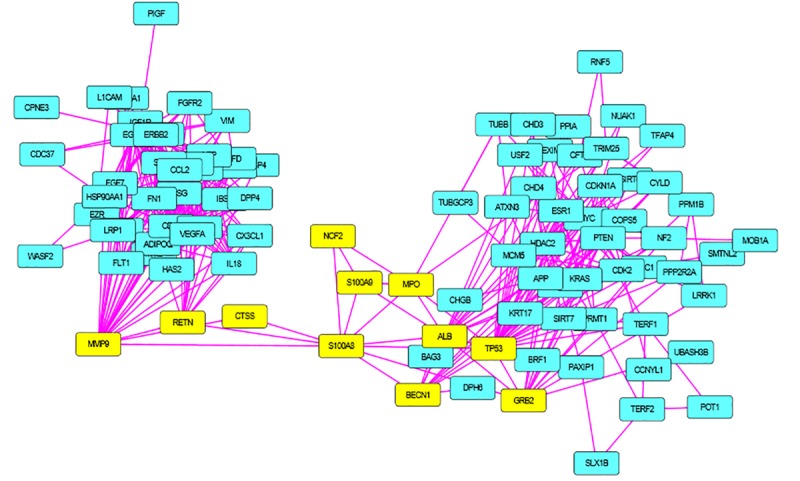
Merged network of Polycystic Ovary Syndrome (PCOS). Combined network of S100A8 integrators with PCOS biomarkers; yellow colored nodes are the first shell interacting proteins with S100A8 that are suspected for cyst migration activity.

**TABLE 1:** Proteins associated with S100A8 in the network.

**Protein ID**	**Name**	**Polycystic Ovary Syndrome (PCOS) relative function**
MMP9	Matrix Metallopeptidase 9	Proteolytic activity on the extracellular matrix (ECM) and involved in leukocyte migration
RETN	Resistin	Promotes chemotaxis in myeloid cells
CTSS	Cathepsin S	Endo protease involved in the removal of unwanted proteins
S100A9	S100 Calcium Binding Protein A9	Potent amplifier of inflammation as well as in cancer development and tumor spread
NCF2	Neutrophil Cytosolic Factor 2	Involved in superoxide generation
MPO	Myeloperoxidase	Produce hypochlorous acid and other toxic intermediates which enhance PMN microbicidal activity
ALB	Albumin	Regulates blood plasma colloid osmotic pressure and acts as a carrier protein for hormones, fatty acids, metabolites, and exogenous drugs
BECN1	Beclin 1	Mediates vesicle-trafficking processes, tumorigenesis, neurodegeneration, and apoptosis
TP53	Tumor Protein P53	Prevents CDK7 kinase activity when associated to CAK complex in response to DNA damage, thus stopping cell cycle progression
GRB2	Growth Factor Receptor Bound Protein 2	Adapter protein involved in the Ras signaling pathway

**FIGURE 3:**
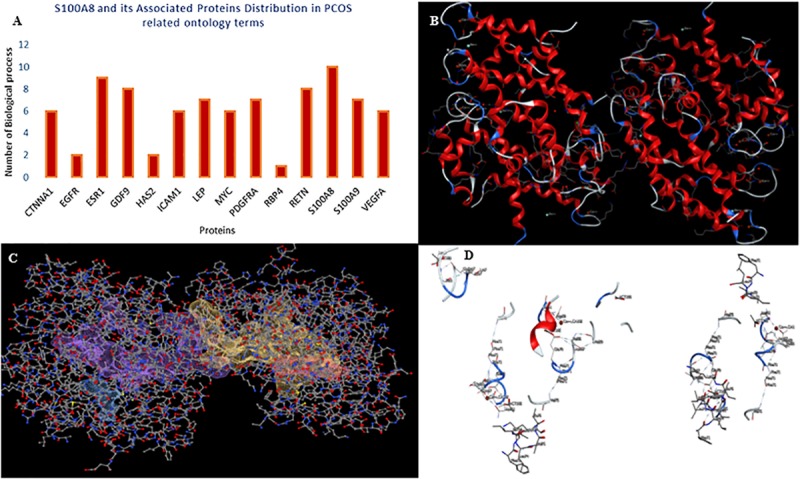
Gene ontology enrichment and structural properties of S100A8. **(A)** Distribution of proteins in polycystic ovary syndrome (PCOS)-related terms in enrichments. **(B)** Homo dimer crystal structure of S100A8—global symmetric view. **(C)** High druggable pockets of S100A8—shown in filled purple, sky blue, yellow, and orange colors. **(D)** Active sites of S100A8.

A total of 246 ontology processes were found within the given significant *p*-value, among them, S100A8 is found in 88 different processes. Particularly, 10 ovulation and maturation-related GO terms with 14 proteins are identified in the enrichment analysis and are listed in Table 2. Apart from S100A8, RETN and S100A9 are found in both networks and also in enriched biological terms with high distribution. ESR1, GDF9, PDGFRA, and LEP are the other proteins found in a greater number of pathways comparatively in the selected terms. Distribution of proteins is given in the graph of Figure 3A.

**TABLE 2:** Enriched terms of S100A8 in Polycystic Ovary Syndrome (PCOS).

**GO term**	**Group *p*-value**	**No. of proteins**	**Associated proteins found**
Ovulation cycle	2.11E-13	9	EGFR, ESR1, GDF9, HAS2, LEP, PDGFRA, RETN, S100A8, S100A9
Female sex differentiation	6.47E-21	12	CTNNA1, ESR1, GDF9, ICAM1, LEP, MYC, PDGFRA, RBP4, RETN, S100A8, S100A9, VEGFA
Ovarian follicle development	6.47E-21	7	CTNNA1, ESR1, ICAM1, MYC, S100A8, S100A9, VEGFA
Ovulation cycle	1.38E-24	9	EGFR, ESR1, GDF9, HAS2, LEP, PDGFRA, RETN, S100A8, S100A9
Ovulation cycle process	1.38E-24	7	ESR1, GDF9, LEP, PDGFRA, RETN, S100A8, S100A9
Development of primary female sexual characteristics	1.38E-24	11	CTNNA1, ESR1, GDF9, ICAM1, LEP, MYC, PDGFRA, RETN, S100A8, S100A9, VEGFA
Gonad development	1.38E-24	11	CTNNA1, ESR1, GDF9, ICAM1, LEP, MYC, PDGFRA, RETN, S100A8, S100A9, VEGFA
Ovarian follicle development	1.38E-24	7	CTNNA1, ESR1, ICAM1, MYC, S100A8, S100A9, VEGFA
Female gonad development	1.38E-24	11	CTNNA1, ESR1, GDF9, ICAM1, LEP, MYC, PDGFRA, RETN, S100A8, S100A9, VEGFA
Regulation of female gonad development	1.38E-24	4	GDF9, RETN, S100A8, S100A9

### Target Compatibility Evaluation

S100A8 is involved in seven GO functions which are positively regulated cyst formation and cancer cell migration. Additionally, S100A8 poses 18 pockets, among them, nine are druggable (score > 0.3) and four shows better cutoff scores (Table 3). Targets with low specificity on small-molecule were identified as poorly druggable targets (Barelier et al., 2010). Here we have found four high scored druggable pockets in the selected S100A8 (Figure 3C). Structural features and active sites of S100A8 are shown in Figures 3B,D, respectively. Due to the positive druggable results, it is considered as a notable target to control PCOS. Considerably, calcium-binding protein (S100A8) acts as a ligand for receptor of advanced glycation end products (RAGE) which is involved in many inflammatory and oncogenic pathways. There is evidence that S100A8 has a growth-promoting effect, and it helps cells to acquire cell migration activity through the RAGE binding pathway (Ghavami et al., 2008). S100A8 causes uteroplacental perfusion deficiency which leads to embryo abortion that supports the competence of our target selection (Sir-Petermann et al., 2002). Structural analysis shows that S100A8 has two helix loop helix Ca2+ binding domains known as EF-hands and exists as a complex with S100A9. Calprotectin is present in 1q21 locus of chromosome 1 in humans and has a molecular weight of 10–12 kDa. During tumor development, chromosomal rearrangements take place in the locus of the S100A8 gene and majorly contribute to the cyst formation in PCOS. Also, serum calgranulin (S100A8 and S100A9) levels are higher in women with PCOS than normal women (Dai and Lu, 2012). This evidently shows that binding of S100A8 with RAGE facilitates the p38 mitogen-activated protein (MAP) kinase signaling through calcium phosphorylation which also governs cyst migration.

**TABLE 3:** Druggability assessment of S100A8 protein.

**Pocket ID**	**Volume A^2^**	**Surface A^2^**	**Drug score**	**Simple score**
P_0	2,694.56	2,620.71	0.81	0.61
P_1	2,652.05	2,822.45	0.81	0.64
P_3	209.65	195.14	0.66	0
P_4	180.41	168.05	0.6	0
P_2	252.39	481.15	0.5	0.14
P_6	166.91	273.52	0.37	0
P_7	165.23	357.95	0.37	0.04
P_5	173.21	283.31	0.35	0
P_8	137.56	211.73	0.33	0
P_9	130.58	204.55	0.28	0
P_10	127.43	196.55	0.27	0
P_11	120.91	239.32	0.27	0
P_14	109.89	180.88	0.26	0
P_12	117.99	208.39	0.25	0
P_13	116.19	209.36	0.22	0
P_15	109.89	259.05	0.16	0
P_16	106.85	216.05	0.15	0
P_17	100.78	249.25	0.14	0

### Construction of RNA Analog Library Using Glucocorticoid Response Element

**FIGURE 4:**
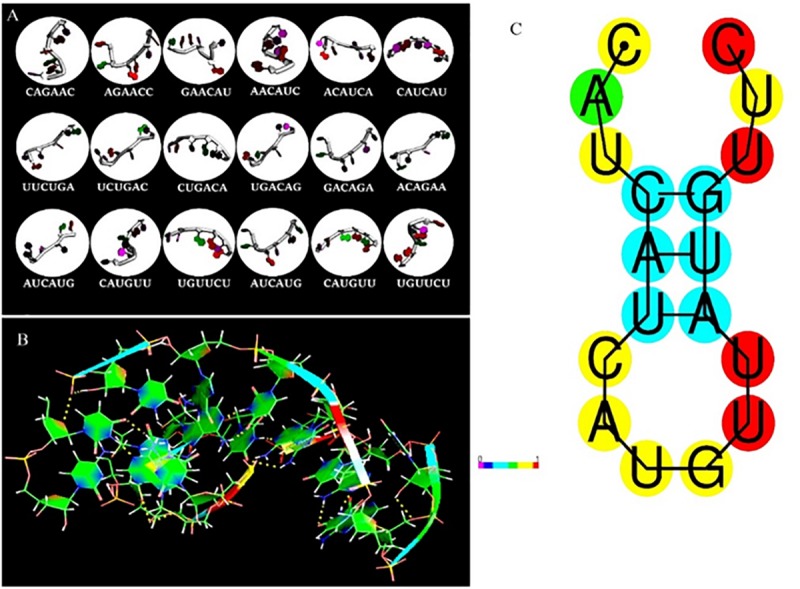
RNA library and structures. **(A)** Library construction by RNA-Lim method using the consensus sequence of glucocorticoid response element (GRE). **(B)** 3D structure of potential aptamer candidate. **(C)** Sequence and physiological structure of aptamer after optimal simulation and folding, and the color of bases indicates their energy levels.

The fragment-based approach of aptamer docking yielded better interaction with S100A8. By the RNA-Lim method, 18 fragments with the consensus sequence of GRE were constructed and used for binding analysis (Figure 4A). Frag6, Frag9, and Frag10 showed better interaction (Table 4) in the active domain of target with minimal global binding energy. Among the three possible conformations, sequence 1 (Figure 4B) shows better thermal stability and lowest energy than the other two sequences. Optimal structure with a folding simulation at physiological pH shows there are three nucleotides at positions 4–7 that make intramolecular base pairing for loop structure (Figure 4C). Energy minimized aptamers are significantly stable, and the aptamers with a binding energy of ≥-40 are optimal in the therapeutical aspect (Pagano et al., 2008). Oligo fragments selected are by their binding ability on the active sites of the target. The compiled 18-mer binds effectively than the fragments. Stability comparison among the newly constructed aptamer sequences is stated in Table 5. Among the three, Apt1 has high stability with a melting temperature of 41.8°C, and also the simulation studies confirmed that it requires the minimum free energy (–27.93 kcal/mol) for hairpin loop formation. In addition, the Apt1 fragment poses low molecular weight (5,327.4 g/mol) comparatively. Aptamers in practice are available in the range of 15–81 nucleotide length with higher molecular weights (Shigdar et al., 2013), but here, the designed is 18-mer with lower molecular weight so the plasma clearance may be faster.

**TABLE 4:** Binding energies of RNA analog fragments with S100A8.

**Fragments**	**Predicted ΔG (kcal/mol)**	**Fragments**	**Predicted ΔG (kcal/mol)**
Frag 1	–16.24	Frag 10	–30.51
Frag 2	–8.55	Frag 11	–14.34
Frag 3	–16.52	Frag 12	–15.66
Frag 4	–51.85	Frag 13	–15.17
Frag 5	–18.46	Frag 14	–11.21
Frag 6	–31.71	Frag 15	–18.70
Frag 7	–16.54	Frag 16	–10.68
Frag 8	–13.17	Frag 17	–21.76
Frag 9	–38.76	Frag 18	–23.71

**TABLE 5:** Aptamer stability comparison.

	**Aptamers**	**GC content (%)**	**Tm (°C)**	**Molecular weight (g/mol)**	**ΔGmax (kcal/mol)**
Apt1	CAUCAUCAUGUUAUGUUC	33.3	41.8	5327.4	–27.93
Apt2	AACAUCACAGAAGACAGA	38.9	37	5504.7	–28.3
Apt3	CUGACAACAUCAAUCAUG	38.9	36.6	5395.5	–29.04

### Interaction, Inhibition, and Stability Studies

**FIGURE 5:**
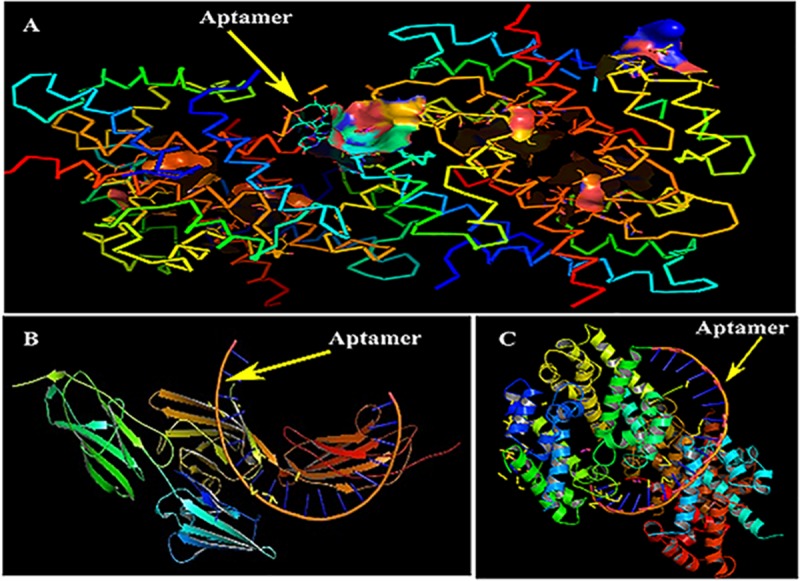
Mechanism of inhibition and docking with RNA aptamer. **(A)** Aptamer bases binding at the active sites of S100A8. **(B)** Binding pose of aptamer on receptor of advanced glycation end products (RAGE) protein. **(C)** Optimal binding pose of aptamer on the S100A8.

Primarily, RAGE being the receptor for S100A8 was docked to confirm for its binding ability in the domain, which may affect the binding of the designed aptamer. As a result of protein–protein docking analysis, Arg 114 residue at A domain of RAGE interacting with Gln 44 residue at H domain of S100A8 is found as the most active interaction. To test the comparison of binding interactions, RAGE was docked with GRE, which resulted in a binding energy of –24.38, comparatively higher than its binding with the designed aptamers (–46.33) that is shown in Table 6; this infers that the designed nucleotide aptamer also binds efficiently at the S100A8 binding domain of RAGE (Figures 5B,C). In parallel, interactions of S100A8 with GRE and S100A8 with the designed aptamer were inspected to find the competency, which was found as –22.11 and –45.32 energy levels, respectively. The designed aptamer binds efficiently in the active dimer of the target (Figure 5A).

**TABLE 6:** Docking results.

**Protein**	**Target**	**Binding site**	**Global energy**
RAGE	S100A8	Arg A 114 → Gln H44	–25.75
RAGE	GRE	Arg B 203 → U_14,_ Arg B 228 → G_17_	–24.38
S100A8	GRE	Asn D 61 → C_6,_ Ala B 1 → U_13_	–22.11
RAGE	Aptamer	• Try B 118 → A_13,_ Arg B 216 → A_13_	–46.33
		• Arg B 218 → G_10,_ Asn B 25 → U_16_	
		• Gln B 24 → U_16_	
S100A8	Aptamer	• Lys B 36 → U_9,_ Lys F 48 → U_17_	–45.32
		• Ser H 86 → A_13,_ Asp C 32 → G_10_	
		• Lys B 18 → G_10,_ Lys B 21 → G_10_	

### Anti-cell Migration Assay on MCF-7 Cell Line

**FIGURE 6:**
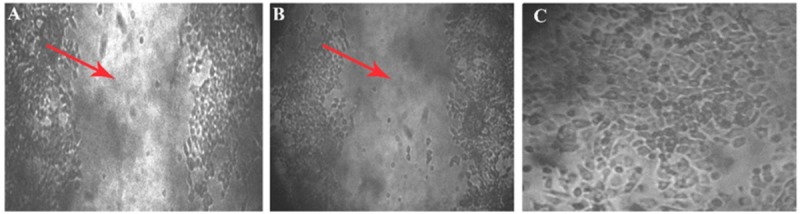
Wound scratch assay. Cell migration studies by wound healing assay on MCF7 cell line. Leitz labovert FS inverted microscope was used to view the cell migration. **(A)** A 4 mm scratch with a sterile tip at 0 h, and the wound is pointed at with an arrowhead in the 10 × magnified illustration. **(B)** Ten times magnified view of aptamer incubated (4 h) cell line. No cell migration was observed after 4 h of aptamer incubation. **(C)** Fifty times magnification of scratch in control plate (cell migration seen) after the fourth hour.

Within 4 h of scratch, development of closure was seen in the control (which does not have aptamer), the wounded area has turned into a normal layer when compared with the initial image of well. In the aptamer well, there is no cell migration observed even after the fourth hour of incubation, it was confirmed in the images of 0 and 4 h of wounded well (Figure 6).

## Conclusion

From the network analysis, S100A8 is identified as a targetable protein to control PCOS. The druggable property of the target was validated by topological measures. S100A8 acts as a ligand for RAGE to promote cell migration in cancers and PCOS conditions. GRE inhibits S100A8 by competitive binding at the minimal level through a feedback mechanism. Additionally, S100A9 and resistin were also found along with S100A8 as associative proteins. We adopted a computational method to develop an RNA aptamer and designed 18 oligos based on the consensus sequences of GRE, which binds to both RAGE and S100A8. In addition to the computational studies, the cell line studies proved the anti-migration activity of the designed aptamer at minimal dose delivery with Lipofectamine 2000. The newly designed 18mer effectively stopped the cancer cell migration through dual action, and it is identified as a potential therapeutic to control PCOS and cancers.

## Data Availability Statement

The raw data supporting the conclusions of this article will be made available by the authors, without undue reservation, to any qualified researcher.

## Author Contributions

All authors listed have made a substantial, direct and intellectual contribution to the work, and approved it for publication.

## Conflict of Interest

The authors declare that the research was conducted in the absence of any commercial or financial relationships that could be construed as a potential conflict of interest.
