# Supplementary material for: Protein Network Studies on PCOS Biomarkers With S100A8, Druggability Assessment, and RNA Aptamer Designing to Control Its Cyst Migration Effect
Source: Front Bioeng Biotechnol. 2020 May 13;8:328. doi: 10.3389/fbioe.2020.00328 (PMC7238949; doi:10.3389/fbioe.2020.00328)
Supplement: Supplementary file 2 [file Image_1.pdf]

Supplimetary figures

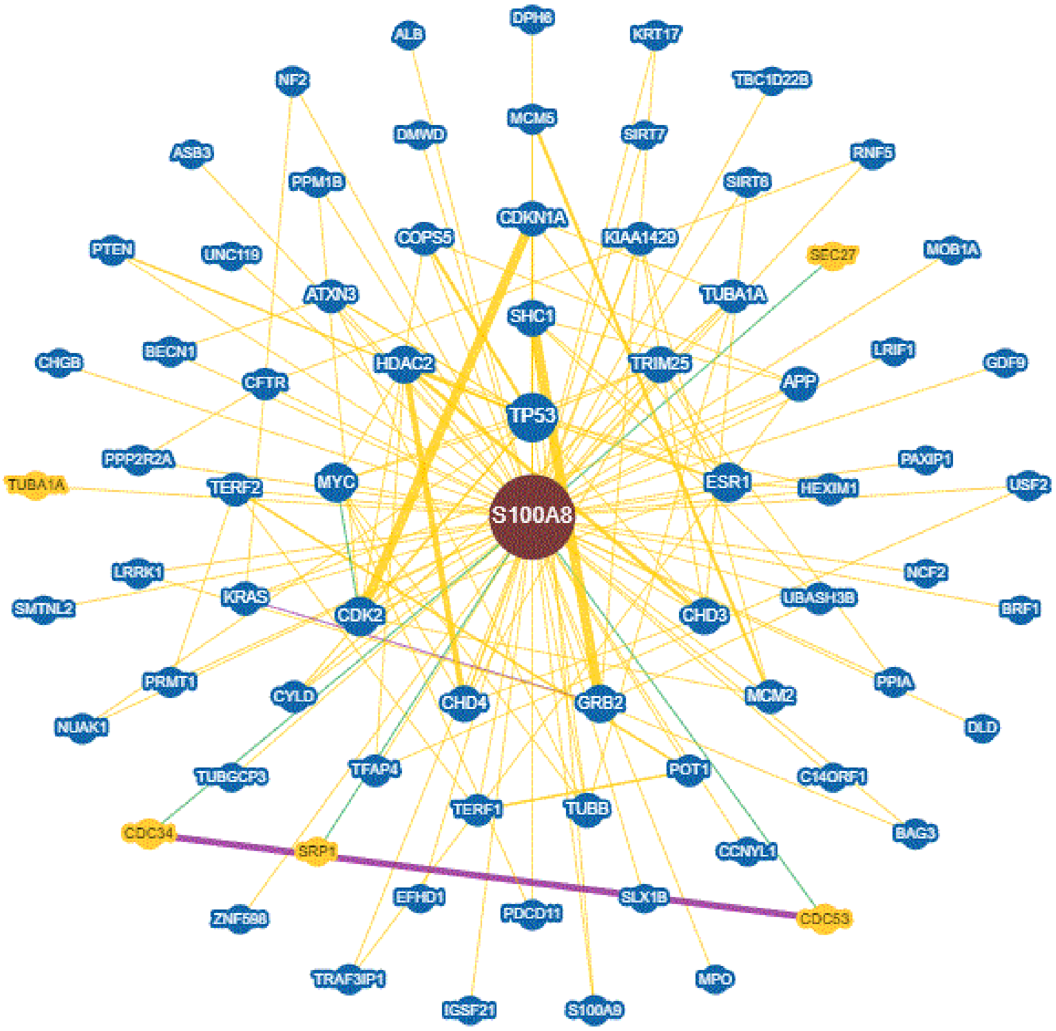

Figure 1. S100A8 interaction network from Biogrid Repository

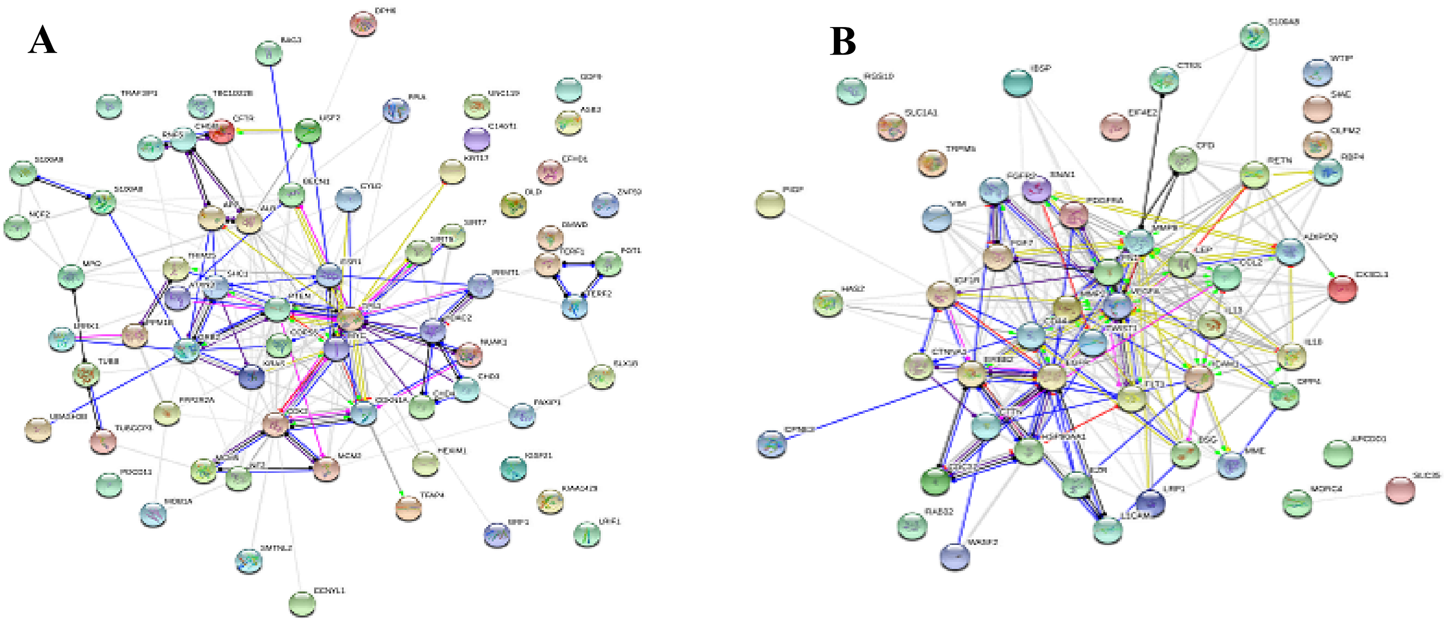

Figure 2. STRING Network of S100A8 (A), network of Biomarkers (B)

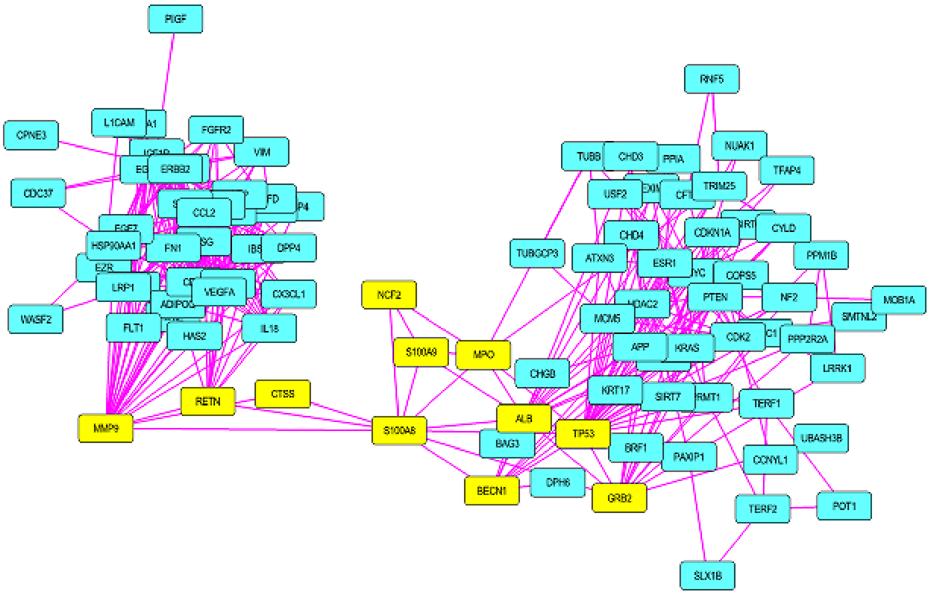

Figure 3. Merged Network

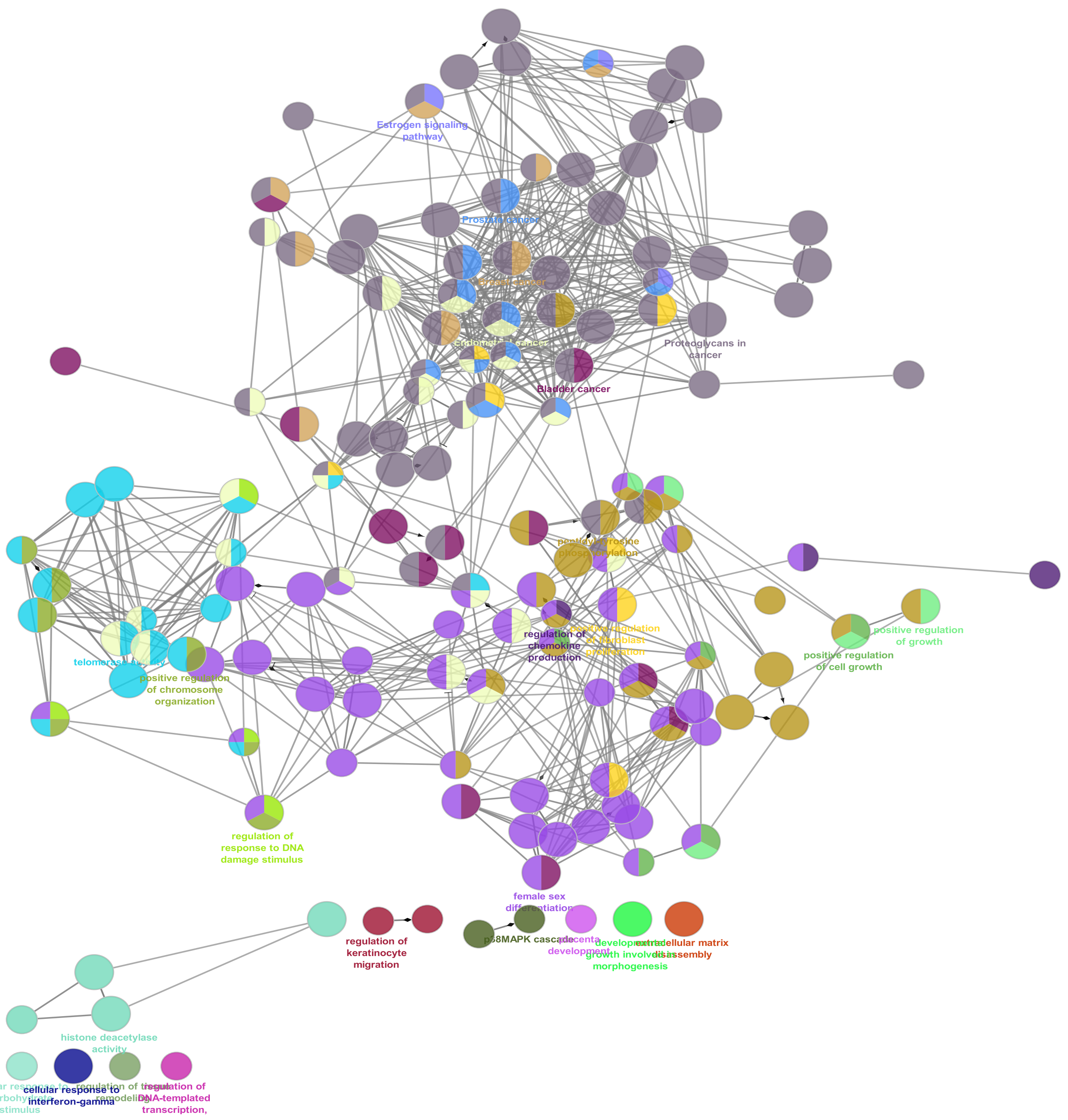

Figure 4.ClueGO Results- Enrichment Clusters

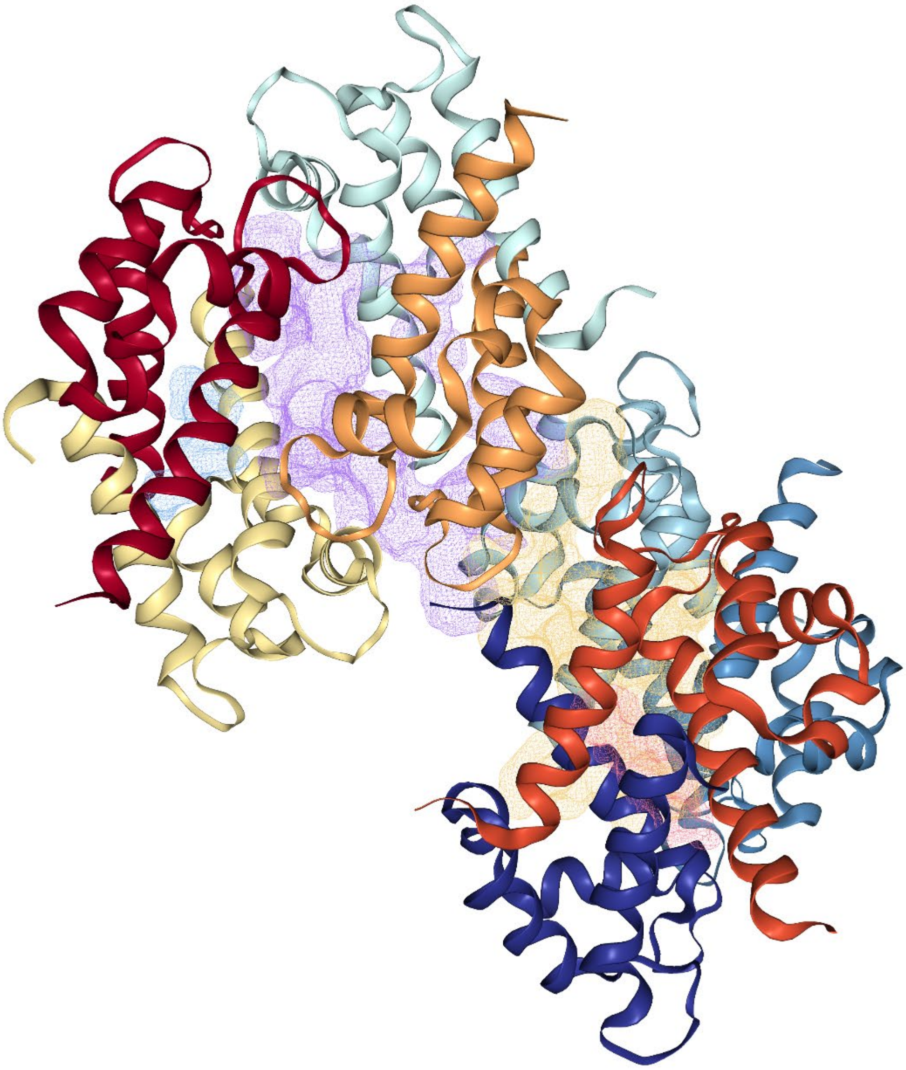

Figure 5. Pocket analysis of S100a8
